# Splanchnic flow analysis in 4D flow MRI: scan-rescan reproducibility of flow and image quality

**DOI:** 10.1007/s00261-025-05238-7

**Published:** 2025-10-15

**Authors:** Paul Ruben Roos, Maarten E. Tushuizen, Jos J. M. Westenberg, Hildo J. Lamb

**Affiliations:** Leiden University Medical Center, Leiden, Netherlands

**Keywords:** 4D flow MRI, Flow analysis, Splanchnic circulation, Reproducibility, Portal vein, End-stage liver disease

## Abstract

**Introduction:**

Assessing the splanchnic circulation may increase understanding and guide management of hepatic disorders. The purpose of this study is to evaluate the scan-rescan reproducibility of abdominal 4D Flow MRI in the splanchnic circulation, to assess the reproducibility of portal venous fractional flow change (FFC), and to compare outcomes healthy volunteers to a patient group with end-stage liver disease (ESLD).

**Methods:**

Ten healthy volunteers (aged 24 ± 3 years) and five patients (aged 62 ± 10 years) with ESLD underwent non-contrast abdominal 4D Flow MRI with respiratory navigator gating twice, with repositioning in between. Flowrates, velocities and cross-sectional area were assessed in abdominal aorta and eight splanchnic vessels. Signal-to-noise ratio (SNR), vessel-sharpness and -length were measured in common portal vein and FFC were calculated.

**Results:**

No significant scan-rescan differences were found in any outcome measures, except for peak-flow in superior mesenteric vein. Intraclass correlation coefficient (ICC) was good to excellent (0.70–0.93) for flowrates and velocities for most vessels, except for some measurements in the hepatic artery, splenic vein and splenic artery. ICC of cross-sectional area was fair (0.53–0.57) or insignificant for most vessels. SNR and vessel-length had excellent ICC (0.96–0.98), but vessel-sharpness only fair ICC (0.59) and FFC was not reproducible in healthy volunteers. FFC was significantly lower in patients than in healthy volunteers by −0.52, while portal vein length was significantly higher by 7.3 mm.

**Conclusion:**

Scan-rescan reproducibility of a 4D Flow MRI acquisition that was optimized in-house for assessing the splanchnic blood flow circulation is good to excellent for flow assessment and maximum velocity in large vessels with high velocities, but not for cross-sectional area and mean velocity. Measurements in smaller vessels and vessels with lower velocities are less reproducible. Fractional flow change was not reproducible in healthy volunteers in the current study and should be assessed in a larger cohort. However, it was indicative for patients with end-stage liver disease.

**Supplementary Information:**

The online version contains supplementary material available at 10.1007/s00261-025-05238-7.

## Introduction

The splanchnic circulation, which refers to the blood flow of the abdominal gastrointestinal organs, is a complex vascular system responsible for the blood supply and drainage of various abdominal organs such as the liver, spleen, stomach and intestine [1]. It plays a critical role in maintaining adequate perfusion to these organs, supporting vital functions such as digestion, absorption, and detoxification. Dysregulation of this system, for instance erroneous blood flow or hypertension, can have significant clinical consequences. Blood flow and blood pressure are closely related and are both required for adequate organ function. For example, in patients with cirrhosis, portal hypertension can impede the drainage of venous blood, producing a reversal of blood flow and may lead to ascites and life-threatening varices [1, 2]. Assessing the splanchnic flow, particularly in the portal and mesenteric veins, can increase the understanding and guide the management of gastrointestinal and hepatic disorders.

Traditional evaluations of splanchnic flow like ultrasound and invasive catheter measurement are limited by the complexity of blood flow, the challenge of accessing deep vessels and the risks associated with catheterization. Emerging techniques such as noninvasive 4D Flow MRI have shown great promise in overcoming these challenges and quantifying blood flow in abdominal vessels throughout the cardiac cycle. This noninvasive MRI technique may allow for better management of patients by reducing the need for (repeated) invasive procedures. Specifically, for the assessment of risk for gastroesophageal varices, 4D Flow MRI based fractional flow change (FFC) in the portal vein has been suggested as an alternative to invasive esophagogastroduodenoscopy, which carries risks such as sedation-related complications and patient discomfort [3].

While 4D Flow MRI has been well established in large arteries and cardiac settings, its application to the abdominal setting is novel [4]. The vasculature presents unique challenges, such as a wide variety of flow velocities and a large amount of respiratory motion. The small vessels require a higher spatial resolution, which lowers the signal-to-noise ratio (SNR) due to the inherent techniques of MRI [5]. This reduced SNR can result in less accurate measurements of blood flow and may limit the ability to fully visualize or quantify complex flow patterns in smaller abdominal vessels. To overcome these limitations, researchers and manufacturers have developed specific abdominal 4D Flow sequences that optimize spatial and temporal resolution, but these are not as widely available as regular cartesian k-space filling [6, 7]. Understanding the reproducibility of general flow measurements and FFC in healthy volunteers and a patient group at risk for portal hypertension will be crucial for validating splanchnic 4D Flow MRI as a diagnostic tool.

We hypothesize that abdominal 4D Flow MRI with cartesian k-space filling can reproducibly quantify blood flow in the splanchnic circulation, particularly the portal and mesenteric veins which are critical for assessing complications of hepatic disorders. Additionally, we aim to investigate the reproducibility of FFC based on 4D Flow MRI measurement, which is particularly important for determining the clinical utility of FFC. With this study, we aim to pave the way for validation studies in different patient populations.

## Methods

### Acquisition

Ten healthy volunteers (*N* = 10) underwent MRI scanning on two occasions with repositioning between sessions. No contrast agent was used and no dietary restrictions were imposed. Scanning was conducted on a 3 T MRI scanner (Elition X, Philips Healthcare, Best, The Netherlands) with software release 5.6. The scanning protocol was executed during both sessions and included a 4D Flow MRI scan that was in-house optimized for assessing the abdominal blood flow circulation. This scan used velocity encoding (venc) of 50 × 50 × 100 cm/s in the anterior-posterior, right-left and feet-head direction, respectively. Additionally, the 4D Flow MRI scan was respiratory-gated using navigator gating of the hemidiaphragm, with a weighted gating protocol [8]. This protocol initiated with filling center of k-space (i.e., CENTRA spiral) using a 5 mm navigator window and extended to 10 mm after acquiring 40% of k-space. Scan parameters are given in Table 1.

**Table 1:** Abdominal 4D flow MRI scan parameters

	4D Flow sequence
FoV [mm^3^]	250 × 381 × 150
Acquired resolution [mm^3^]	2.5 × 2.5 × 2.5
Reconstructed resolution [mm^3^]	1.5 × 1.5 × 1.5
Temporal resolution [ms]	39.2
Slice gap [mm]	−1.0
TR [ms]	4.9
TE [ms]	3.2
Flip Angle [°]	10
Sense factor	3/1.5
Segmentation factor	2
Respiratory compensation	Navigator gating with Weighted Gating
Cardiac phases	15
Venc [cm/s]	50 × 50 × 100 (RL x AP x FH)

*FoV* Field of View, *TE* Echo time, *TR* Repetition time, *Venc* Encoding velocity

After informed consent, five patients with end-stage liver disease underwent the same 4D Flow MRI scan. Patients were in the hospital after admittance to liver transplant screening and had no history of vascular surgeries or procedures such as transjugular intrahepatic portosystemic shunt (TIPS) or contraindications for MRI. Patients all fasted for at least four hours before MRI acquisition.

The study was conducted in accordance with the Declaration of Helsinki. The study in healthy volunteers was approved by the Medical Ethics Committee Leiden The Hague Delft (P18.034).The MRI examination in the patients was approved by the Medical Ethics Committee Leiden The Hague Delft (P23.078). All participants gave informed consent.

### Data analysis

Abdominal 4D Flow MRI data was analyzed using CAAS MR Solutions 5.3 (Pie Medical Imaging B.V., Maastricht, The Netherlands) by a single researcher with 4 years of experience. Flow analysis was performed in three arteries and 5 veins: the abdominal aorta (superior to the celiac trunk), the common hepatic artery (CHA), the splenic artery (SA), the splenic vein (SV), the superior mesenteric vein (SMV), the portal vein (PV) and the left and right portal vein branches. The analysis included visualizing the vessels with the 4D Artery workflow of beforementioned software, placing perpendicular reformatting planes on each vessel for flow velocity mapping and drawing vessel contours manually on the planes, enabling the calculation of flows, velocities and cross-sectional area. Flow volume per heartbeat and peak flow rate was assessed per vessel. At peak flow rate, vessel contour area and mean velocity within the contour area were assessed. Peak flow rate was chosen, as the higher velocities result in a higher velocity to noise ratio, possibly improving vessel visibility and thereby delineation.

The fractional flow change (FFC) in the portal vein was calculated using the following equation:

$$\:FFC=\:\frac{PV-(SMV+SV)}{SMV+SV}.$$

A FFC below zero indicates hepatofugal flow, which is an indirect measurement of blood that is shunted away from the liver [4]. The proximal splenic vein is measured to account for anatomical differences of the drainage of the left gastric vein into the splenic vein.

An axial slice of the magnitude image of the fast gradient echo that included the common portal vein was chosen to measure the signal-to-noise ratio (SNR), and a magnitude image of the phase contrast was used to assess the vessel sharpness (Supplemental Fig. 1A). Two regions of interest (ROI) were drawn: an oval ROI covering the portal vein and a circular ROI in the air outside of the body. SNR was defined as the mean intensity value in the ROI of the portal vein divided by the standard deviation of the intensity signal in the ROI of the air.

A line perpendicular through the portal vein was defined and an intensity profile of the line was exported. Vessel sharpness was measured as the average of the slope of the line profile at either side of the vessel. The slopes were calculated by dividing the difference in minimal and maximal signal intensity by the difference in distance (Supplemental Fig. 1B).

The vessel length of the common portal vein was identified by first segmenting the portal vein in the velocity visualization in CAAS MR Solutions 5.3. Then, the centerline of the vessel was calculated and planes perpendicular to the centerline were placed in the proximal and distal common portal vein, close to the convergence of the splenic and superior mesenteric vein or the branching into the left portal vein branch, respectively. The distance of the centerline between these two planes was measured three-dimensionally.

Spleen size was calculated with an ellipsoid formula [9]. The maximum length, width and depth were measured on a coronal scout MRI scan and an axial DIXON scan. The three parameters were then multiplied together with pi divided by 6, to yield the spleen size.

In five healthy volunteer cases, flow analysis was performed twice in the super mesenteric vein, splenic vein and portal vein. The resulting flow parameters, including FFC, were used to assess intra-observer variability of the flow analysis, including the manual delineation of the vessel contours.

### Statistical analysis

Differences in flow volumes and peak flow rates, velocities and cross-sectional area between scan and rescan MRI were assessed. For each individual vessel, scan-rescan differences in flow were assessed as well. Statistical significance of these differences was assessed with paired sample t-tests and evaluated by Bland-Altman plots. Agreement between scans was evaluated through the calculation of the intraclass correlation coefficient (ICC) and the coefficient of variation (CoV). CoV was defined as the standard deviation between the difference of scan and rescan measurements, divided by the mean of the parameter [10]. It must be noted that CoV can be misleading for means close to zero [11]. ICC between scan and rescan measurements were calculated as single rater absolute agreement with a two-way random effects model. ICCs with significant p-values were classified as poor (< 0.5), fair (> 0.5 and < 0.7), good (> 0.7 and < 0.85) and excellent (> 0.85). Correlation between portal vein flow and the sum of flow in the portal vein branches was assessed with Pearson correlation test. Differences between measurements in healthy volunteers and patients were assessed with t-tests or Mann-Whitney U tests, depending on the normality of the distribution of the data. Where applicable, means, standard deviations and p-values are given. Statistical power of correlation tests and FFC comparison were calculated.

## Results

### Scan-rescan

**Fig. 1:**
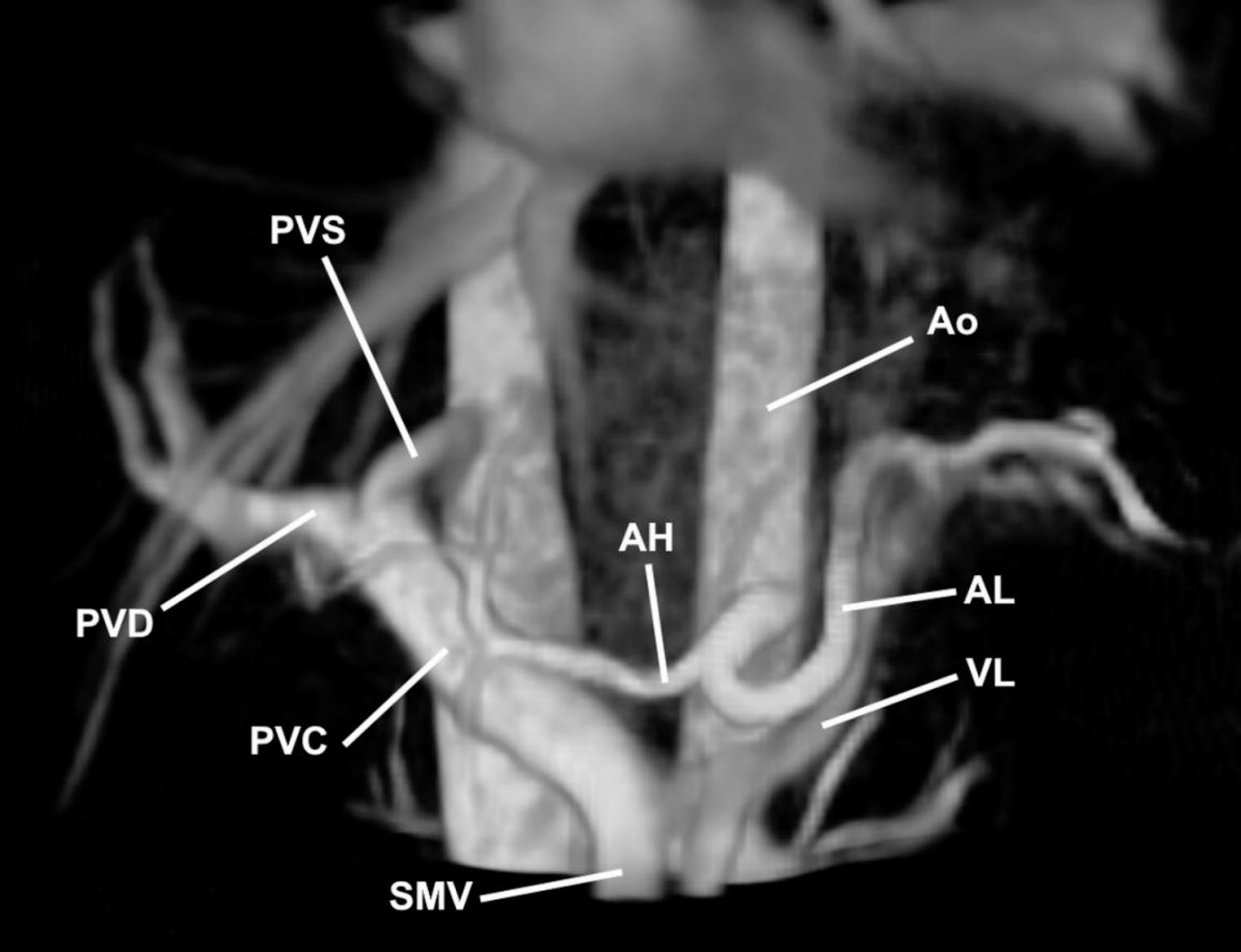
Overview of splanchnic blood vessels measured with abdominal 4D Flow MRI in a healthy volunteer (21 years old, male). *Ao* Abdominal Aorta, *AL* Splenic Artery (Arteria Lienalis), *AH* Hepatic Artery (Arteria Hepatica), *VL* Splenic Vein (Vena Lienalis), *VMS* Superior Mesenteric Vein (Vena Mesenterica Superior), *VPC* Common Portal Vein (Vena Porta Communis), *VPS* Left Portal Vein Branch (Vena Porta Sinistra), *VPD* Right Portal Vein Branch (Vena Porta Dextra)

**Fig. 2:**
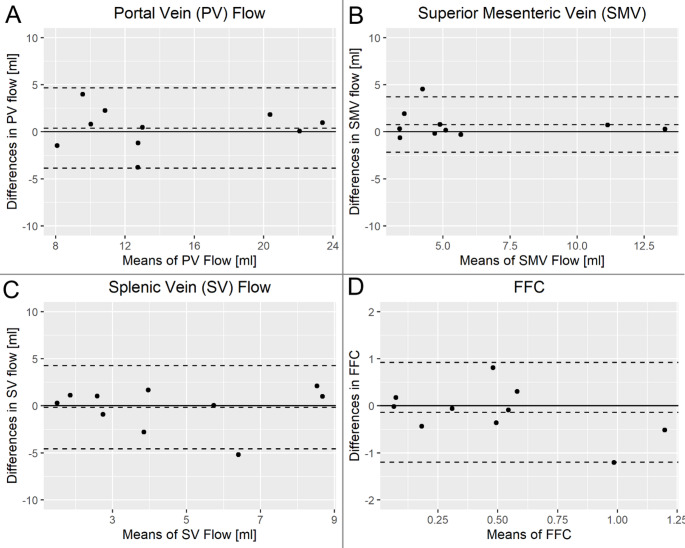
Bland-Altman plots of scan-rescan differences in flow volumes of (**A**) portal vein, (**B**) splenic vein, (**C**) superior mesenteric vein and (**D**) fractional flow change (FFC)

Healthy volunteers were aged 24 ± 3 years (70% male). Acquisition of 4D Flow sequences took around 10–14 min. All eight blood vessels could be identified and visualized in all ten volunteers (Fig. 1). No significant differences between scan and rescan measurement of FFC, SNR, vessel sharpness or vessel length were found (Table 2; Fig. 1D). Scan-rescan ICC was excellent (0.96–0.98) for SNR and vessel length, 0.59 for vessel sharpness, and insignificant for FFC. Likewise, CoV was 16%, 6% and 29% for SNR, vessel length and vessel sharpness, respectively, and 110% for FFC. In two scans, the FFC reached below 0 (−0.013 and − 0.034). These were scans of two different individuals and different scan sessions. Bland-Altman plots (Fig. 2) of scan-rescan differences of FFC and the flow volumes of the three vessels that FFC calculation is based on, show no statistical trends.

**Table 2:** Scan-rescan differences in abdominal 4D flow MRI parameters

	Mean		Paired t-test		ICC		CoV
Measurement	Scan	Rescan	Difference	p-value	ICC (95% CI)	p-value	[%]
FFC	0.42 (± 0.34)	0.56 (± 0.55)	−0.13	0.44	0.31 (−0.37-0.77)	0.17	110%
SNR	269 (± 200)	288 (± 228)	20	0.20	0.98 (0.91–0.99)	< 0.01	16%
Vessel sharpness	59 (± 33)	50 (± 17)	9	0.25	0.59 (0.03–0.88)	0.02	43%
Vessel length	46 (± 9)	47 (± 9)	−0.5	0.62	0.96 (0.84–0.99)	< 0.01	6%

*FFC* Fractional Flow Change, *SNR* Signal-to-noise ratio

No significant scan-rescan difference in flow volume, peak flow rate, cross-sectional area at peak flow rate and mean velocity at peak flow rate was found in any vessel, except for the peak flow rate in the superior mesenteric vein (Table 3). This flow rate was found to be slightly higher (on average 1.8 ml/s) in the first scan (*p* = 0.03), but ICC was 0.80 and CoV only 25%. The reduction in flow volume in the superior mesenteric vein was not significant. Flow volume per heart beat had good to excellent (0.70–0.93) scan-rescan ICC for all vessels, and was lowest for the splenic artery and vein (ICC = 0.70 and 0.71, and CoV = 41% and 49%, respectively). Peak flow rate ICC was high to excellent (0.72–0.89) for all vessels, except for splenic vein, for which it was fair (0.64). The comparison of flow rates between the common portal vein and the two branches demonstrated an excellent correlation (r^2^ = 0.94, *p* < 0.001).

**Table 3:** Scan Rescan differences per vessel

	Means		Paired t-test		ICC		CoV
	Scan	Rescan	Mean diff.	p-value	ICC (CI)	p-value	[%]
Flow volume [ml]							
Ao	59.9 (± 22.0)	60.4 (± 17.1)	−0.5	0.87	0.89 (0.62–0.97)	< 0.01	16
AH	2.5 (± 1.9)	2.5 (± 1.8)	0.0	0.98	0.81 (0.40–0.95)	< 0.01	46
AL	5.2 (± 2.7)	4.9 (± 2.5)	0.3	0.63	0.70 (0.15–0.92)	0.01	41
VL	4.5 (± 2.9)	4.7 (± 2.9)	−0.2	0.83	0.71 (0.16–0.92)	0.01	49
VMS	6.3 (± 3.4)	5.6 (± 3.6)	0.8	0.14	0.90 (0.64–0.97)	< 0.01	25
VPC	14.5 (± 5.7)	14.1 (± 5.6)	0.4	0.58	0.93 (0.76–0.98)	< 0.01	15
VPS	5.1 (± 2.6)	4.5 (± 2.0)	0.7	0.16	0.82 (0.41–0.96)	< 0.01	27
VPD	8.0 (± 4.2)	7.2 (± 3.9)	0.8	0.17	0.92 (0.70–0.98)	< 0.01	21
Peak flow [ml/s]							
Ao	234.0 (± 82.0)	223 (± 64.8)	11.3	0.33	0.89 (0.64–0.97)	< 0.01	15
AH	4.4 (± 3.1)	4.2 (± 2.8)	0.2	0.80	0.81 (0.39–0.95)	< 0.01	43
AL	8.7 (± 4.4)	7.8 (± 3.6)	0.9	0.38	0.72 (0.23–0.92)	0.01	36
VL	6.7 (± 3.8)	6.4 (± 3.4)	0.4	0.73	0.64 (0.03–0.90)	0.02	48
VMS	9.8 (± 4.3)	8.0 (± 4.4)	1.8	0.03	0.80 (0.27–0.95)	< 0.01	25
VPC	20.1 (± 6.3)	19.4 (± 6.5)	0.6	0.60	0.83 (0.47–0.96)	< 0.01	19
VPS	7.5 (± 3.0)	6.2 (± 2.4)	1.3	0.07	0.72 (0.15–0.93)	0.01	27
VPD	11.4 (± 5.2)	10.2 (± 5.0)	1.2	0.19	0.86 (0.55–0.96)	< 0.01	24
Area at peak flow [mm^2^]							
Ao	258 (± 61)	272 (± 50)	−14	0.24	0.79 (0.40–0.94)	< 0.01	13
AH	42 (± 20)	42 (± 14)	0	0.99	0.47 (−0.25-0.84)	0.09	43
AL	59 (± 15)	50 (± 21)	9	0.11	0.57 (0.02–0.87)	0.02	28
VL	61 (± 31)	61 (± 21)	0	1.00	0.33 (−0.42-0.79)	0.18	51
VMS	113 (± 47)	92 (± 36)	21	0.13	0.53 (−0.3-0.85)	0.03	37
VPC	174 (± 22)	168 (± 43)	6	0.57	0.53 (−0.12-0.86)	0.05	20
VPS	110 (± 21)	101 (± 23)	9	0.21	0.54 (−0.06-0.87)	0.04	20
VPD	132 (± 35)	126 (± 31)	5	0.45	0.81 (0.41–0.95)	< 0.01	16
Mean velocity at peak flow [cm/s]							
Ao	90.9 (± 23.6)	82.3 (± 19.2)	8.6	0.02	0.84 (0.29–0.96)	< 0.01	11
AH	10.7 (± 4.5)	10.0 (± 5.7)	0.6	0.65	0.75 (0.28–0.93)	< 0.01	35
AL	14.8 (± 6.5)	16.0 (± 6.2)	−1.2	0.41	0.77 (0.34–0.94)	< 0.01	28
VL	11.1 (± 2.9)	10.3 (± 4.0)	0.8	0.52	0.43 (−0.24-0.82)	0.09	35
VMS	8.9 (± 2.0)	8.5 (± 2.3)	0.3	0.68	0.30 (−0.42-0.77)	0.20	30
VPC	11.4 (± 2.5)	11.5 (± 2.0)	−0.1	0.86	0.46 (−0.26-0.84)	0.09	21
VPS	6.7 (± 2.1)	6.0 (± 1.2)	0.6	0.18	0.68 (0.14–0.92)	0.01	21
VPD	8.6 (± 2.9)	7.8 (± 2.5)	0.8	0.24	0.73 (0.27–0.93)	< 0.01	24

*Ao* Abdominal Aorta, *AH* Hepatic Artery (Arteria Hepatica), *AL* Splenic Artery (Arteria Lienalis), *VL* Splenic Vein (Vena Lienalis), *VMS* Superior Mesenteric Vein (Vena Mesenterica Superior), *VPC* Common Portal Vein (Vena Porta Communis), *VPS* Left Portal Vein Branch (Vena Porta Sinistra), *VPD* Right Portal Vein Branch (Vena Porta Dextra)

Reproducibility of cross-sectional area at peak flow rate was statistically significant in most vessels, but not all (Table 3). The hepatic artery and the splenic vein had non-significant ICC (*p* = 0.09 and 0.18, and CoV = 43% and 51%, respectively). ICCs for cross-sectional area were good (0.79–0.81) in aorta and right vena porta branch, but only fair (0.53–0.57) in other vessels. The scan-rescan ICC for mean velocity at peak flow was statistically significant in all vessels but three, namely splenic vein, superior mesenteric vein and the portal vein (*p* = 0.09, 0.20 and 0.09, respectively). CoV was maximally 35% in these three vessels. In the left portal vein branch, ICC was 0.68. In the other vessels, the three arteries and the right portal vein branch, ICC was good (0.73–0.84). Statistical power was over 0.93 for excellent correlations, and on average 0.80 for good correlations.

Intra-observer variability in portal vein, superior mesenteric vein and splenic vein was excellent for flow (ICC = 0.99, CoV = 10%), peak flow (ICC = 0.99, CoV = 8%) and, area at peak flow (ICC = 0.89, CoV = 22%), and good for mean velocity at peak flow (ICC = 0.82, CoV = 14%). FFC itself had an excellent ICC of 0.91, but a CoV of 52%.

### Patients with end-stage liver disease

**Fig. 3:**
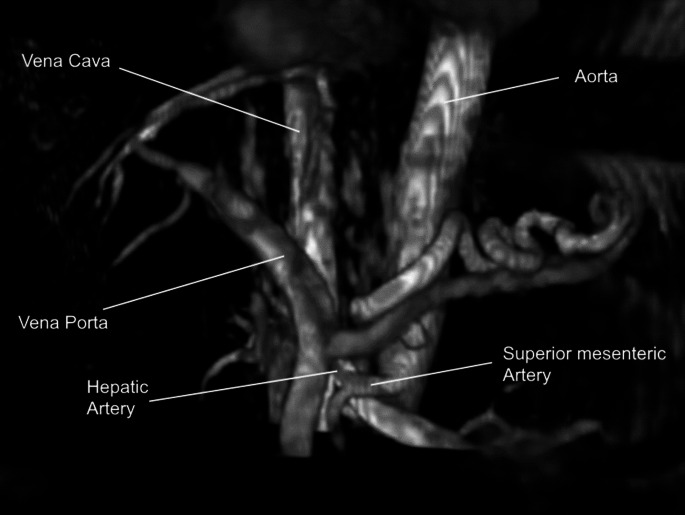
Splanchnic blood vessels in a patient (male, 68 years old) with end-stage liver disease. An anatomical variety where the hepatic artery arises from the superior mesenteric artery instead of the coeliac trunk

Patients were aged 62 ± 10 years old, weighed 85 ± 13 kg, and four were male. All eight vessels could be identified in all five patients (see one example in Fig. 3). Two patients presented with an anatomical variety where the hepatic artery originates from the superior mesenteric artery instead of the coeliac trunk. No large portosystemic shunts were discovered for any of the patients. Mean measurements in the patients and healthy volunteers are given in Table 4. FFC was on average − 0.03 (± 0.09) in patients and 0.49 (± 0.45) in healthy volunteers, which was statistically significantly lower (*p* < 0.01, power = 0.77). Common portal vein vessel length was also significantly higher in patients with 53.9 mm (± 5.0) versus 46.6 mm (± 9.1, *p* = 0.03). No statistically significant differences in flow volume were found between patients and healthy volunteers. Spleen size was on average significantly higher (*p* = 0.01) in patients (644 ± 471 ml) than in healthy volunteers (221 ± 87 ml), with two patients presenting splenomegaly.

**Table 4:** Abdominal 4D flow parameters in splanchnic circulation in patients with end-stage liver disease versus healthy volunteers

	Patients	Healthy volunteers	T-test	
			Mean difference	*p*-value
FFC	−0.03 (± 0.09)	0.49 (± 0.45)	−0.52	< 0.01
SNR	185 (± 76)	279 (± 209)	−93	0.12
Vessel sharpness	42 (± 15)	54 (± 26)	−12	0.19
Vessel length	53.9 (± 5.0)	46.6 (± 9.1)	7.3	0.03
Flow volume [ml]				
Ao	51.7 (± 9.7)	60.2 (± 19.2)	−8.5	0.19
AH	3.3 (± 2.4)	2.5 (± 1.8)	0.8	0.53
AL	7.1 (± 1.8)	5.0 (± 2.5)	2.0	0.07
VL	4.6 (± 1.2)	4.6 (± 2.8)	0.0	0.99
VMS	8.1 (± 1.8)	5.9 (± 3.4)	2.1	0.08
VPC	12.1 (± 1.8)	14.3 (± 5.5)	−2.2	0.15
VPS	4.8 (± 1.3)	4.8 (± 2.3)	0.0	0.96
VPD	7.8 (± 2.6)	7.6 (± 4.0)	0.2	0.90

*FFC* Fractional Flow Change, *SNR* Signal-to-noise ratio, *Ao* Abdominal Aorta, *AH* Hepatic Artery (Arteria Hepatica), *AL* Splenic Artery (Arteria Lienalis), *VL* Splenic Vein (Vena Lienalis), *VMS* Superior Mesenteric Vein (Vena Mesenterica Superior), *VPC* Common Portal Vein (Vena Porta Communis), *VPS* Left Portal Vein Branch (Vena Porta Sinistra), *VPD* Right Portal Vein Branch (Vena Porta Dextra)

## Discussion

### Scan-rescan

In this study, measurements of flow volume, peak flow rate, cross-sectional area and the mean velocity at peak flow rate from 4D Flow MRI in splanchnic circulation were shown to be reproducible in most vessels. FFC was not significantly different between scan sessions in healthy volunteers, and significantly reproducible in patients with end-stage liver disease. Reproducibility of SNR and vessel length was excellent, but reproducibility of vessel sharpness was only fair. Significant differences in FFC and common portal vein length was found between healthy volunteers and patients with end-stage liver disease.

Flow volume had excellent scan-rescan ICC for aorta, superior mesenteric vein and the portal vein and the right portal vein branch. ICC was good for all other vessels, but CoV was 41%−49% for the liver artery and the splenic artery and vein. The higher CoV for flow parameters with a mean close to zero, as is true for smaller vessels with lower velocities, is to be expected due to the nature of CoV [11]. These three vessels are also the smallest in diameter. Similarly, the peak flow rate in these three vessels had the highest CoV of 36%−48% compared to the other vessels. The size of these vessels are likely at the limit of what can reproducibly be measured with the voxel size in the 4D Flow MRI sequence used.

The cross-sectional area at peak flow rate, which is typically in the cardiac phase with the highest velocity-to-noise ratio and therefore the best image quality, was only reproducible with a high ICC in the abdominal aorta and the right portal vein branch. ICCs were even insignificant for the hepatic artery and splenic vein, for which the CoVs were above 43% as well. Vessels with lower velocities may be more difficult to delineate due to the lower velocity-to-noise ratio [5]. Additionally, similar to the measurement of flow volume and peak flow rate, the vessels might be too small to accurately delineate due to the voxel size of 4D Flow MRI and the partial volume effect [12]. Correcting this effect has been shown to be feasible in small intracranial vessels and may also be feasible in abdominal applications [13].

Mean velocity at peak flow rate was not statistically different in scan-rescan, but ICCs were not significant in splenic vein, superior mesenteric vein and common portal vein, and CoVs were above 30% for hepatic artery, splenic vein and superior mesenteric vein. Maximum velocities from phase contrast MRI are known to be underestimated, mostly due to limitations of the spatiotemporal resolution [5]. This can be particularly apparent in small vessels, such as the splenic vein [12]. Additionally, lower velocities can reduce the accuracy of vessel delineation due to the low velocity-to-noise ratio in situations with a higher encoding velocity setting (venc), thereby altering the mean velocity measurement [14, 15]. Furthermore, in larger veins such as the superior mesenteric vein and the common portal vein, helical or vortical flow may occur that may include lower velocities.

### FFC

Although flow measurement in the portal vein, superior mesenteric vein and splenic vein had high to excellent reproducibility, the FFC measurement in healthy volunteers itself had no significant scan-rescan ICC. The minor differences in flow in these individual vessels may summate to the larger differences in the FFC measurement. In patients with end-stage liver disease, the negative flow rate may be more apparent and the FFC may be more reproducible, which warrants further investigation in a study with larger cohorts. In two of the volunteer scans, the FFC was below zero, but this was also not reproducible. In patient scans, the FFC was below zero on average, which would be expected with end-stage liver disease. The FFC measurement was also significantly lower in patients than in healthy volunteers, suggesting it may have a diagnostic value. The FFC in the patient scans with a negative FFC (mean = −0.10) was also lower than in the two negative FFC measurements in the volunteers (mean = −0.02), meaning a more significant component of hepatofugal flow. Literature suggests no cut-off value in negative FFC for diagnostic purposes yet, but it is a promising method that warrants further investigation in a larger cohort study [4]. Besides technical feasibility, FFC should be assessed in a larger cohort study with direct comparison to other standards such as Hepatic Venous Pressure Gradient (HVPG) or Doppler Ultrasound.

Spleen size can impact splanchnic blood flow: an increased spleen size is related to an increase in portal vein blood flow volume and cross-sectional area, but not to portal pressure, azygos blood flow or portal vein blood flow velocity [16, 17]. Spleen size is unlikely to influence FFC, as an increase in splenic vein flow would be paired with an increase in portal vein flow, but this remains to be investigated. In this study, patients were found to have an increased spleen size compared to healthy volunteers, as would be expected with end-stage liver disease, depending on the etiology [18].

### MRI acquisition and analysis

In this study, a cartesian 4D Flow MRI sequence was employed with adapted velocity encoding optimized per direction (i.e. venc 100 cm/s in FH direction and 50 cm/s in RL and AP direction, respectively). The generalizability of this sequence was specifically chosen for our study, as this type of sequence is readily available on most clinical MRI-scanners and is not vendor specific. While the current sequence has been optimized in-house for the abdominal setting, this sequence can be implemented on most MRI-scanners which would benefit translation to other centers to yield similar results.

To mitigate respiratory motion artefacts, navigator gating with weighted gating was utilized [8]. This sequence incorporated a “contrast-enhanced timing-robust angiography (CENTRA) spiral readout”, which uses cartesian readout in a pseudo-spiral pattern. The combination of weighted gating and the CENTRA spiral readout facilitated the acquisition of the center of k-space with effective respiratory motion compensation, while also reducing the overall acquisition time. However, no respiratory motion compensation method is completely effective and motion artifacts could still impact flow measurements of smaller and more mobile vessels. Previous studies have used employed alternative methods, such as stack-of-stars readouts or single breath-hold spiral readouts [6, 7, 19]. Cartesian 4D Flow MRI, in different varieties, is available on more MRI systems than the other readout methods and this study confirms that it can be used accurately in abdominal applications.

Due to the presence of both venous and arterial flow, and therefore low and high velocities of interest, a single encoding velocity setting results in a decrease of SNR in venous flow and possible aliasing artefacts in arterial flow [20]. The latter can be corrected for in post-processing, but these correction techniques are not always available or robust [21]. The use of two encoding velocities (dual-venc) may alleviate these limitations at the cost of greatly increased acquisition time or data undersampling [22–24]. These dual-venc sequences were not readily available and scanning twice with different encoding velocities was not used to not overburden patients and volunteers beyond the already long scan times involved in this study. Further developments in both acquisition and post-processing may improve abdominal 4D Flow MRI in the coming years.

Assessment of flow parameters and image quality was performed in multiple software packages. Automatic and semiautomatic approaches for vessel contouring were available and considered, but ultimately not used because of inaccuracies in small vessels and at low velocities. The entire analysis including contouring was performed by only one person, thereby mitigating inter-observer variability, but intra-observer variability is still present. This variability was assessed in five healthy volunteers and was excellent for all parameters except mean velocity at peak flow, for which it was good. Previous studies in contouring of larger vessels and heart valve found low coefficients of variation (< 5%), while CoV was higher in our study with lower and smaller vessels [25]. It is worth noting that CoV becomes unreliable at means closer to zero, so a higher CoV is expected in the flow parameters in this study [11]. This is apparent in the CoV of the intra-observer variability of FFC (52%), which has a mean very close to zero, while the ICC was excellent (0.91).

Healthy volunteers had no presence or prior presence of any disease related to the measurements performed in this study. In four out of five patients, a small amount of ascites was present, but no associated artefacts were discovered. No paracentesis was performed before MRI acquisition.

### Limitations

The cohort sizes of both patients and volunteers are limited to just investigate the technical feasibility of the presented abdominal 4D Flow MRI methodology. However, for derived metrics like FFC and some specific flow parameters in smaller vessels with lower velocities, the coefficient of variation was high. The power of the correlation tests was over 0.93 for excellent correlations, but around 0.80 for good correlations. The comparison of the FFC had a power of 0.77. A larger cohort size can increase the power of the statistical analysis to decrease the chance for type II errors, so significant correlations will not be accidentally missed. The results of the metrics in smaller vessels with lower velocities in this study are indicative of the variation and reproducibility of the flow parameters, but should be confirmed in future studies prior to full acceptance.

4D Flow MRI with cartesian sampling has been validated extensively in the past, both in vitro as well as in vivo, and compared to other modalities such as Doppler ultrasound, in various settings [26–29]. Additional validation was not performed in this study.

Although patients were instructed to fast for at least four hours prior to scanning, no instructions regarding diet were given to healthy volunteers. Postprandial flow in the superior mesenteric vein, is increased and peaks around 40 min after meal intake, but may take a longer time to return to resting state [30]. It is likely that the significant decrease in peak flow rate in the superior mesenteric vein is part of the final period of postprandial response. Given the long scan sessions of 2 h total, it is likely that volunteers ate before the MRI sessions, explaining the reduction in peak flow rate in the SMV over time. Dietary restrictions should be considered for future imaging studies on splanchnic blood flow.

Both patients and volunteers were only positioned and repositioned in the supine head-first position, as was per standard protocol for abdominal MRI. Acquiring 4D Flow MRI with the subject in prone position may yield different results, which has yet to be investigated. Furthermore, including different positions in reproducibility assessments may yield a more in-depth analysis of real-world clinical scenarios.

## Conclusion

Scan-rescan reproducibility of a 4D Flow MRI acquisition that was optimized in-house for assessing the splanchnic blood flow circulation is good to excellent for flow assessment and maximum velocity in large vessels with high velocities, but not for cross-sectional area and mean velocity. Measurements in smaller vessels and vessels with lower velocities are less reproducible. Fractional flow change was not reproducible in healthy volunteers in the current study and should be assessed in a larger cohort. However, it was indicative for patients with end-stage liver disease.

## Supplementary Information

Below is the link to the electronic supplementary material.

Supplementary file1 (DOCX 526 kb)

## Data Availability

No datasets were generated or analysed during the current study.
