# Supplementary material for: Splanchnic flow analysis in 4D flow MRI: scan-rescan reproducibility of flow and image quality
Source: Abdom Radiol (NY). 2025 Oct 15;51(5):2685–95. doi: 10.1007/s00261-025-05238-7 (PMC13061781; doi:10.1007/s00261-025-05238-7)
Supplement: Supplementary file 1 — Supplementary file1 (DOCX 526 kb) [file 261_2025_5238_MOESM1_ESM.docx]

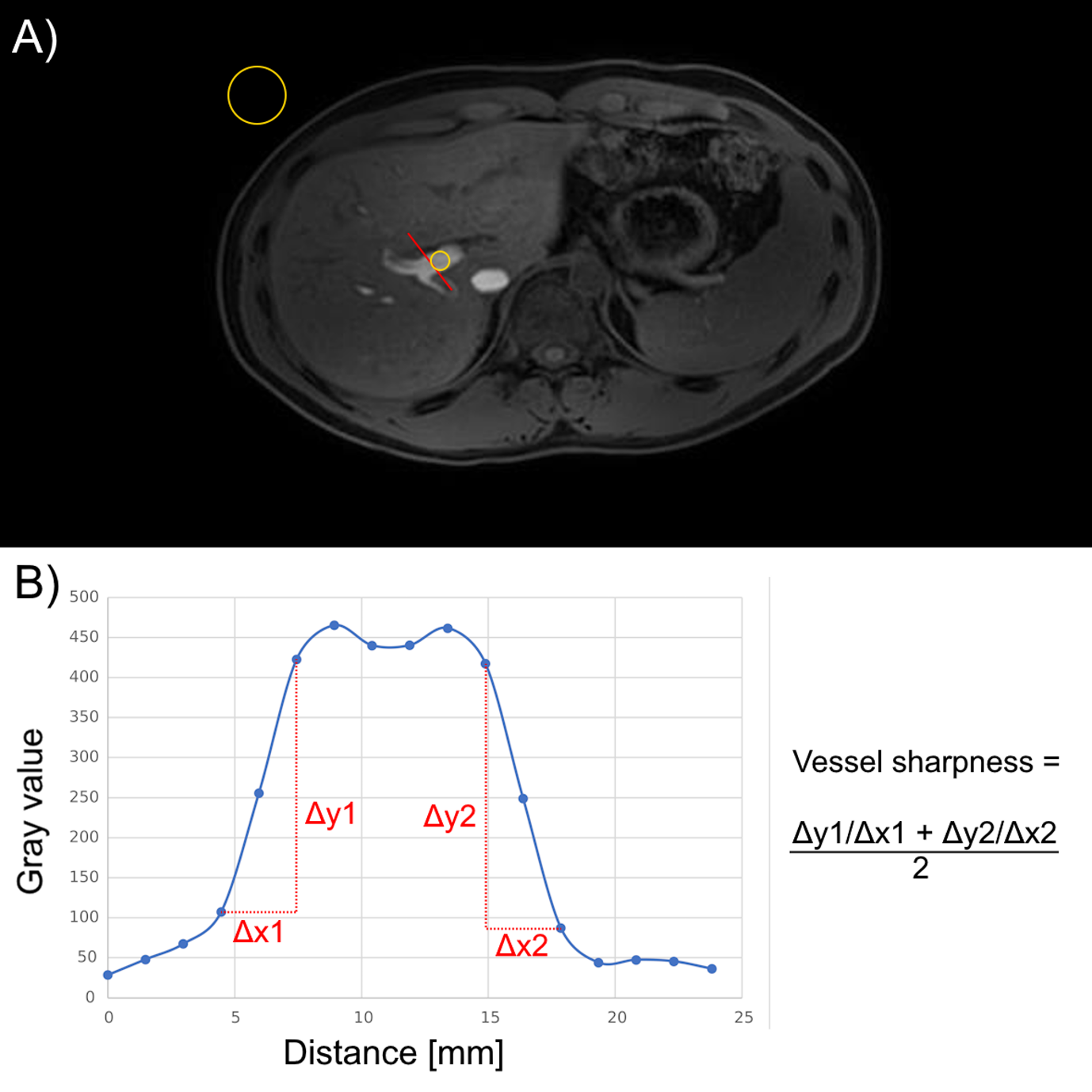


Supplemental Fig. 1: Illustration of vessel sharpness and SNR measurement. Dixon MRI was used only for illustrative purposes, actual measurements were performed on 4D Flow MRI data. A) MRI image with red line to indicate where line-profile was measured and yellow ROI circles to indicate where SNR measurement was taken. B) Line profile with measurements and calculation of vessels sharpness.
